# Eco-physiological adaptation of dominant tree species at two contrasting karst habitats in southwestern China

**DOI:** 10.12688/f1000research.2-122.v2

**Published:** 2013-11-25

**Authors:** Shouren Zhang, Dayong Fan, Qian Wu, Hui Yan, Xinwu Xu

**Affiliations:** 1 State Key Laboratory of Vegetation and Environmental Change, Institute of Botany, the Chinese Academy of Sciences, Beijing, 100093, China; 2 Graduate University of the Chinese Academy of Sciences, Beijing, 100093, China

**Keywords:** karst habitat; photosynthesis; chlorophyll fluorescence; stomatal sensitivity; water potential

## Abstract

The purpose of this study was to investigate the eco-physiological adaptation of indigenous woody species to their habitats in karst areas of southwestern China. Two contrasting forest habitats were studied: a degraded habitat in Daxiagu and a well-developed habitat in Tianlongshan, and the eco-physiological characteristics of the trees were measured for three growth seasons. Photosynthetic rate (Pn), stomatal conductance (gs), and transpiration rate (Tr) of the tree species in Daxiagu were 2-3 times higher than those in Tianlongshan under ambient conditions. However, this habitat effect was not significant when measurements were taken under controlled conditions. Under controlled conditions, Pn, gs, and Tr of the deciduous species were markedly higher than those for the evergreen species. Habitat had no significant effect on water use efficiency (WUE) or photochemical characteristics of PSII. The stomatal sensitivity of woody species in the degraded habitat was much higher than that in the well-developed habitat. Similarly, the leaf total nitrogen (N) and phosphorus (P) contents expressed on the basis of either dry mass or leaf area were also much higher in Daxiagu than they were in Tianlongshan. The mass-based leaf total N content of deciduous species was much higher than that of evergreen species, while leaf area-based total N and P contents of evergreens were significantly higher than those of deciduous species. The photosynthetic nitrogen- and phosphorus-use efficiencies (PNUE and PPUE) of deciduous species were much higher than those of evergreens. Further, the PPUE of the woody species in Tianlongshan was much higher than that  of the woody species in Daxiagu.

The results from three growth seasons imply that the tree species were able to adapt well to their growth habitats. Furthermore, it seems that so-called “temporary drought stress” may not occur, or may not be severe for most woody plants in karst areas of southwestern China.

## Introduction

Karst topography features soluble bedrock, which is usually carbonate rock such as limestone or dolomite ^1^. Karst topography is characterized by a very slow formation of soil from the carbonate rock, leading to low water retention capacity ^1, 2^. China’s karst topography is located mainly in the southwestern region, which is also characterized by diversified landscape types and a dense population. Deforestation was once one of the most serious environmental problems in China’s karst region, and was mainly due to fuel wood production, agricultural expansion, and livestock husbandry. This land use has caused the ecosystem of the karst region to degrade beyond its already poor condition. As a result, many woodlands have degraded to rocky desert in many of the karst regions in southwest Guizhou Province. Karst ecosystems are very fragile. If destroyed, the soil regeneration process is slow, and thus karst ecosystems are only restored gradually, if at all. Precipitation is sufficient in this region, but the woody plants are supposedly subjected to temporary water stress. This water stress results from low soil water-holding capability, and the high leakiness of limestone rock ^2, 3^. Numerous studies have simulated the above environments, and the data generated has shown that plants are suffering from conditions of temporary water-stress ^2, 3^. In most of these studies, potted seedlings were used, and the experiments were conducted under controlled environments (e.g. greenhouses). It is risky to extrapolate the situation for mature tree species in their natural conditions from data from potted seedlings under controlled conditions. So far, reports on woody plant growth and physiological response to temporary water deficiency in the karst field areas have been sparse. This is particularly so for mature woody plants. Investigating how woody plants respond functionally to differently degraded habitats will help understand the adaptive mechanisms that these indigenous species have to their habitats. This will also help in optimizing the selection of tree species for forest ecosystem restoration in karst regions.

For our study, we selected two types of forests with contrasting karst habitats in the west and southwest of Guizhou Province: a well-developed secondary deciduous and evergreen broad-leaved mixed forest at Tianlongshan Mountain, and a severely degraded forest dominated by spare deciduous woody species at Daxiagu. We made *in situ* measurements of eco-physiological traits for the dominant tree species in three consecutive growth seasons (2007–2009) to address the following questions:

1) Compared with tree species in the well-developed forest habitat, are the eco-physiological traits down-regulated for the tree species in the degraded karst habitat?

2) Is there a difference in stomatal sensitivity between tree species in the two contrasting forests?

3) How do nutrients affect the eco-physiological characteristics of the tree species in the two contrasting forests?

## Materials and methods

### Study areas

For the purpose of making comparisons of how the tree species adapt to their own different karst habitats, we designed the experiment at two contrasting mature forest habitats. Tianlongshan, located in the west of Guizhou Province, has a relatively well-developed karst secondary forest. Daxiagu, located in the southwest of Guizhou Province, has a severely degraded karst forest.

Tianlongshan is located about 10 km south of Puding County in western Guizhou Province (26°15′N, 105°44′E) at an altitude of about 1200 m. This region is dominated by a humid monsoon climate. The mean annual temperature is 15.1°C ^4^. The mean annual precipitation is 1398 mm, and 60–70% of the rainfall events occur during the growth season between May and October ^4^. Tianlongshan has a well-developed secondary evergreen and deciduous broad-leaved mixed forest growing in lime yellow soil. Tree heights range from 3–7 m.

Daxiagu is located 20 km southwest of the town of Huajiang in southwestern Guizhou Province (25°42′N, 105°35′E) at an altitude of about 900 m. This area has a warm temperate climate and a mean annual temperature of 18.4°C ^4^. The mean annual precipitation is 1100 mm, with 83% of this precipitation occurring during the growth season between May and October. Vegetation in this region is characterized by sparsely distributed secondary deciduous trees and shrubs on bare rocks.

Field investigations and collections of tree leaf samples in these two sites did not require specific permits. For the purposes of our work, collaboration with local (provincial) universities or research institutes was required. The locations of our field investigations are neither privately-owned nor protected lands. The tree species used in our investigations and sampling were not endangered or protected.

The dominant tree species in Tianlongshan and Daxiagu found in this study are listed in Table 1.

**Table 1.** Characteristics of the tree species examined in this study. For leaf phenology, D represents deciduous and E represents evergreen; For location, T represents Tianlongshan and Dx represents Daxiagu.

Species	Leaf phenology	Location	Measurement year
*Platycarya longipes*	D	T	2008, 2009
*Lithocarpus glabra*	D	T	2007,2008,2009
*Celtis sinensis*	D	T, Dx	2008, 2009
*Ligustrum lucidum*	E	T	2007, 2008, 2009
*Quercus aliena* var. *acutiserrata*	D	T	2007, 2008, 2009
*Lindera communis*	E	T	2007, 2008, 2009
*Daphniphyllum oldhami*	E	T	2008, 2009
*Stachyurus obovatus*	E	T	2007, 2008, 2009
*Carpinus pubescens*	D	T	2007, 2008, 2009
*Itea chinensis*	E	T	2007, 2008, 2009
*Zanthoxylum ovalifolium* var. *spinifolium*	D	T	2009
*Ilex chinensis*	E	T	2007
*Alangium chinense*	D	Dx	2008, 2009
*Rhus chinensis*	D	T, Dx	2008, 2009
*Picasma quassioides (D. Don) Benn*	D	Dx	2008, 2009
*Broussonetia papyrifera*	D	Dx	2008, 2009
*Mallotus japonicus* var. *floccosus*	D	Dx	2008, 2009
*Rhamnella franguloides*	D	Dx	2008, 2009
*Viburnum chinshanense*	D	Dx	2008, 2009
*Sapium sebiferum*	D	Dx	2007, 2008, 2009
*Ficus benguetensis*	E	Dx	2007, 2008, 2009
*Melia azedarach* Linn.	D	Dx	2007
*Solanum verbascifolium*	D	Dx	2007
*Mallotus barbatus*	D	Dx	2007
*Vernicia fordii*	D	Dx	2007
*Alchornea davidii*	D	Dx	2007
*Mallotus philippensis*	E	Dx	2007
*Flemingia philippinensis*	D	Dx	2007

### *In situ* photosynthetic gas exchange and chlorophyll *a* fluorescence transient measurements

3–4 trees for each species were randomly (the nearest one every 20–30 m distance) selected from the study sites, and a detached branch from each tree was obtained from the top or middle sunny side (south-facing) of the canopy. Branches were detached using a pair of pruning shears mounted on a 5 m pole. The detached branch was immediately immersed in a water-filled bucket. The end of each branch was re-cut twice under water, ensuring continuity of the xylem conduit. Photosynthetic rate (Pn), stomatal conductance (g _s_), transpiration rate (Tr), and instantaneous water use efficiency (WUE) were measured with new, fully expanded leaves from the detached branches using a LI-6400 photosynthesis system (LI-COR Inc., Lincoln Nebraska, USA). Measurements were conducted around 8:30–13:00 (Beijing Standard Time). Measurement conditions were set at a PAR (photosynthetically active radiation) level of 1000 µmol m ^-2^s ^-1^. Leaf temperature and humidity were at their ambient conditions during the measurement period of June–July 2007 and 2008. During this time, the leaf temperatures were 22–25°C in Tianlongshan and 30–33°C in Daxiagu.

Plant stomatal sensitivity was obtained using Lohammar’s hyperbolic function method ^5– 7^. We measured g _s_-VPD (leaf-air vapor pressure deficiency) curves in July 2009, in which the VPD was manually set from saturated vapor [over 85% of relative humidity (RH)] to the driest vapor (about 5% of RH). Leaf temperature was held at 30°C, and PAR at 1000 µmol m ^-2^s ^-1^. The g _s_-VPD curves were fitted using the modified Lohammar’s function: g _s_ = -m × ln D + b, to estimate stomatal sensitivity (m) ^8^. In this equation, g _s_ and D stand for stomatal conductance and VPD, respectively, and m and b are parameters generated in a least square regression analysis.

Chlorophyll *a* fluorescence transients were measured in the leaves from the branches collected as detailed above for photosynthetic gas exchange measurements using a Handy-PEA portable fluorometer (Hansatech Instruments Ltd., Norfolk, UK). Before measurements were taken, the leaves were darkened for at least 30 min using leaf clips (Hansatech Instruments Ltd., Norfolk, UK). The polyphasic chlorophyll *a*fluorescence transients OJIP [fluorescence levels O: F _o_ (50 μs); J: F _J_ (2 ms); I: F _I_ (30 ms), and P: F _p_=F _m_ (tF _max_)] were analyzed according to the JIP test procedure ^9, 10^. The JIP test procedure has been widely used in studies of eco-physiology and stress physiology ^10^. Several parameters can be derived from the following fluorescence values: 50 μs (Fo, step O), 100 μs (F100), 300 μs (F300), 2ms (step J), 30 ms (step I), and the maximum (Fm, step P), using Biolyzer software (version 3.0.7.2 and available by contacting Dr. Reto Strasser at Reto.Strasser@unige.ch). According to Strasser *et al.* ^9, 10^, the definition of the JIP test parameters used in this study can been summarized as follows: As flux ratios or quantum yields expressed as maximal trapping flux (TRo) to the reaction center of PS II, maximal electron transport flux (ETo) between PS II and PS I, and maximal heat dissipation flux (DIo) of PSII per the light flux absorbed (ABS) by PS II antenna. Φ(Po), maximum quantum yield of primary photochemistry; Φ(Eo), quantum yield of electron transport, probability that an absorbed photon will move an electron into electron transport further than Q _A_^-^; Φ(Do), quantum yield of dissipation; PI(abs), performance index on the basis of light energy absorption, which is responsible for the performance of the electron transport from water to plastoquinone.

Φ(Po)=1–(Fo/Fm)=Fv/Fm=TRo/ABS; Fo=F50 μs, minimal fluorescence at O-step (50 μs); Fm=FP, maximal fluorescence at P-step;

Φ(Eo)=(Fv/Fm)×Ψ(Eo); Ψ(Eo)=(1–V _J_), V _J_=(F _J_-Fo)/(Fm–Fo), F _J_=F2ms, fluorescence at J-step (2ms) of O-J-I-P;

Φ(Do)=1–Φ(Po)=Fo/Fm=DIo/ABS;

PI(abs)=(RC/ABS)×[Φ(Po)/(1–Φ(Po))]×[Ψ(Eo)/(1-Ψ(Eo))]; RC=reaction center, ABS=absorption flux.

### Leaf water potential

The midday leaf water potential (Ψ) was determined for each species at the two habitats using a PSYPRO Water Potential System (Wescor, Inc., USA). The measurements were carried out between 11:00 and 14:00 hours (Beijing Standard Time) in August 2009 on leaves from the same detached branches from which the photosynthesis/fluorescence measurements were taken.

### Leaf nutrient assay

Leaf total N content and soil total organic N content (%) were determined using the Kjeldahl acid-digestion method (Kjeltec 2200 Auto Distillation Unit, Foss, Denmark). Leaf total phosphorus (P) content and soil total P content (%) were analyzed colorimetrically (UV-visible spectrophotometer, UV-2550, Shimadzu Corporation, Japan).

### Statistical analysis

Before the Analysis of Variance (ANOVA) was carried out, all data was examined graphically for the normality of distribution (probability plots for residual analysis), and the homogeneity of variance (scatter plots) using Data Desk (version 6.01, Data Description, Inc., 1996). After examination using the above methods, all data except leaf water potential satisfied the assumption for ANOVA for normal distribution and homogeneity of variance. The data for leaf water potential were log-transformed, and the transformed data satisfied the normal distribution assumption for ANOVA. The effects of habitat (i.e. the severely degraded karst habitat vs. well-developed secondary forest habitat) and life form (i.e. deciduous vs. evergreen) were tested using the two-way ANOVA procedure in Data Desk.

## Results

### Photosynthetic gas exchange

**Figure 1.**
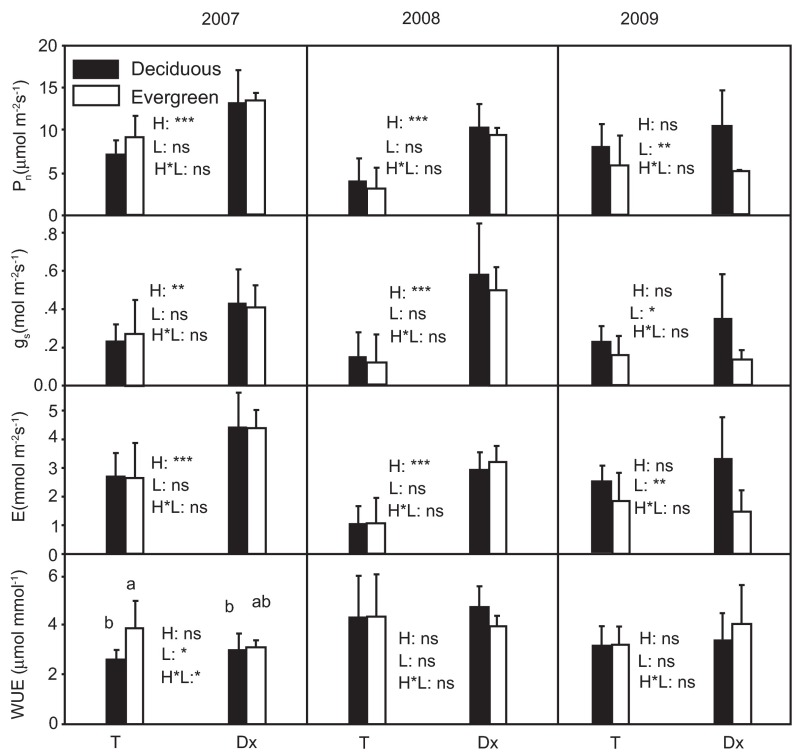
Photosynthetic gas exchange characteristics. Effect of habitat (H), leaf phenology (L), and their interaction (H × L), on Pn, g _s_, E, and WUE (mean ± SD, n = 3–5) of the tree species in two habitats. T and Dx represent the two study sites Tianlongshan and Daxiagu, respectively. Measurements were conducted under ambient conditions in 2007 and 2008, and under controlled conditions in 2009. The significance levels (*** = P < 0.001, ** = P < 0.01,* = P < 0.05, and ns = P > 0.05) were based on ANOVA results.

When measured under their respective ambient conditions in 2007 and 2008 (i.e. ambient temperature and humidity, and PAR set at 1000 µmol m ^-2^s ^-1^), the photosynthetic rate (Pn) across all investigated tree species was much higher in Daxiagu (around 2–3 times, P < 0.001) than in Tianlongshan ( Figure 1). The g _s_ and Tr of the investigated tree species in Daxiagu were also much higher (P < 0.001) than those in Tianlongshan ( Figure 1). However, when measurements were taken under controlled conditions in 2009 (i.e. PAR was set at 1000 µmol m ^-2^s ^-1^, leaf temperature at 30°C, and VPD at 1 KPa), there was no significant habitat effect on Pn ( Figure 1). There was a significant difference in Pn between deciduous and evergreen tree species across all investigated tree species in the two habitats. The Pn of deciduous tree species was significantly higher (P < 0.01) than the Pn of evergreen tree species ( Figure 1). The g _s_ and Tr of the deciduous tree species were also significantly higher than the g _s_ and Tr of evergreen tree species (P < 0.05 and 0.01, respectively). The habitat effect on gs and Tr was not statistically significant ( Figure 1). The effects of leaf phenology (deciduous vs. evergreen) and habitat were not significant for instantaneous water use efficiency (WUE) under either ambient or controlled measurement conditions in either habitat.

Photosynthesis data Photosynthetic traits of the dominant tree species as well as the environmental factors at Daxiagu Tianlongshan were measured in the summer seasons of 2007, 2008 and 2009.

### Chlorophyll fluorescence

**Figure 2.**
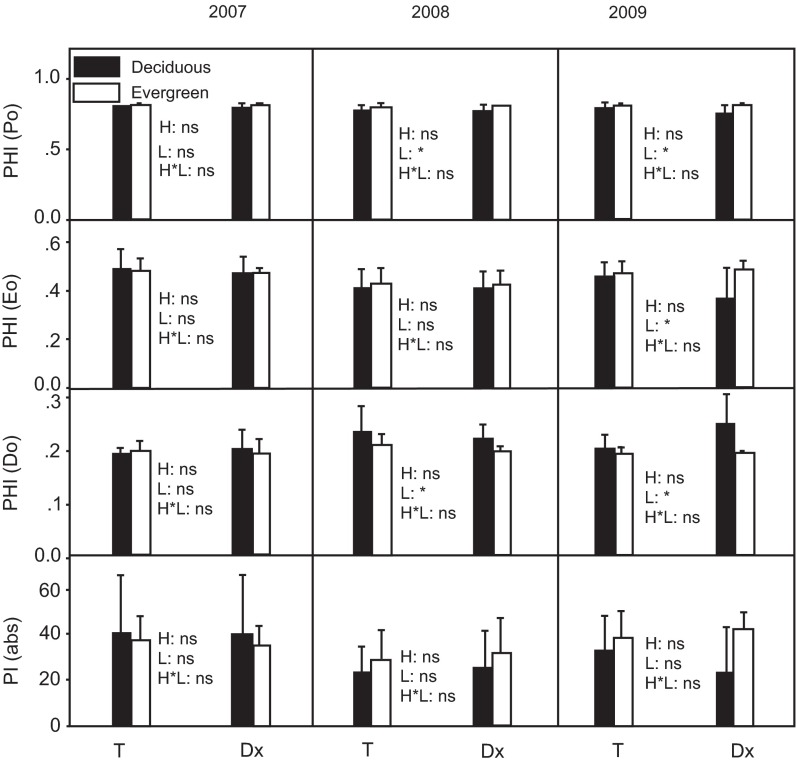
Photochemical characteristics. Effects of habitat (H), leaf phenology (L), and their interaction (H × L), on maximal PSII efficiency (Φ(Po)), quantum yield of PSII electron transport (Φ(Eo)), quantum yield of dissipation Φ(Do), and performance index (PI(abs)) (mean ± SD, n = 3–5) of the tree species in two habitats. See Figure 1 for other explanations.

Leaf phenology and habitat had no significant (P > 0.05) effect on chlorophyll fluorescence parameters in 2007 ( Figure 2). However, the effect of leaf phenology became significant (P < 0.05) for maximal PSII efficiency (Φ(Po)), quantum yield of PSII electron transport (Φ(Eo)), quantum yield of dissipation (Φ(Do)), and the comprehensive parameter for assessing plant’s vitality: performance index (PI(abs)), for all investigated tree species in 2008 and 2009 ( Figure 2). The Φ(Po), Φ(Eo), and PI(abs) of the evergreen tree species were significantly higher than those of the deciduous tree species, while the opposite effect of leaf phenology was seen in Φ(Do).

Chorophyll fluorescence data Chlorophyll fluorescence characteristics of the dominant tree species at Daxiagu and Tianlongshan were measured in the summer seasons of 2007, 2008, and 2009.

### Stomatal sensitivity, leaf water potential, and specific leaf area

**Figure 3.**
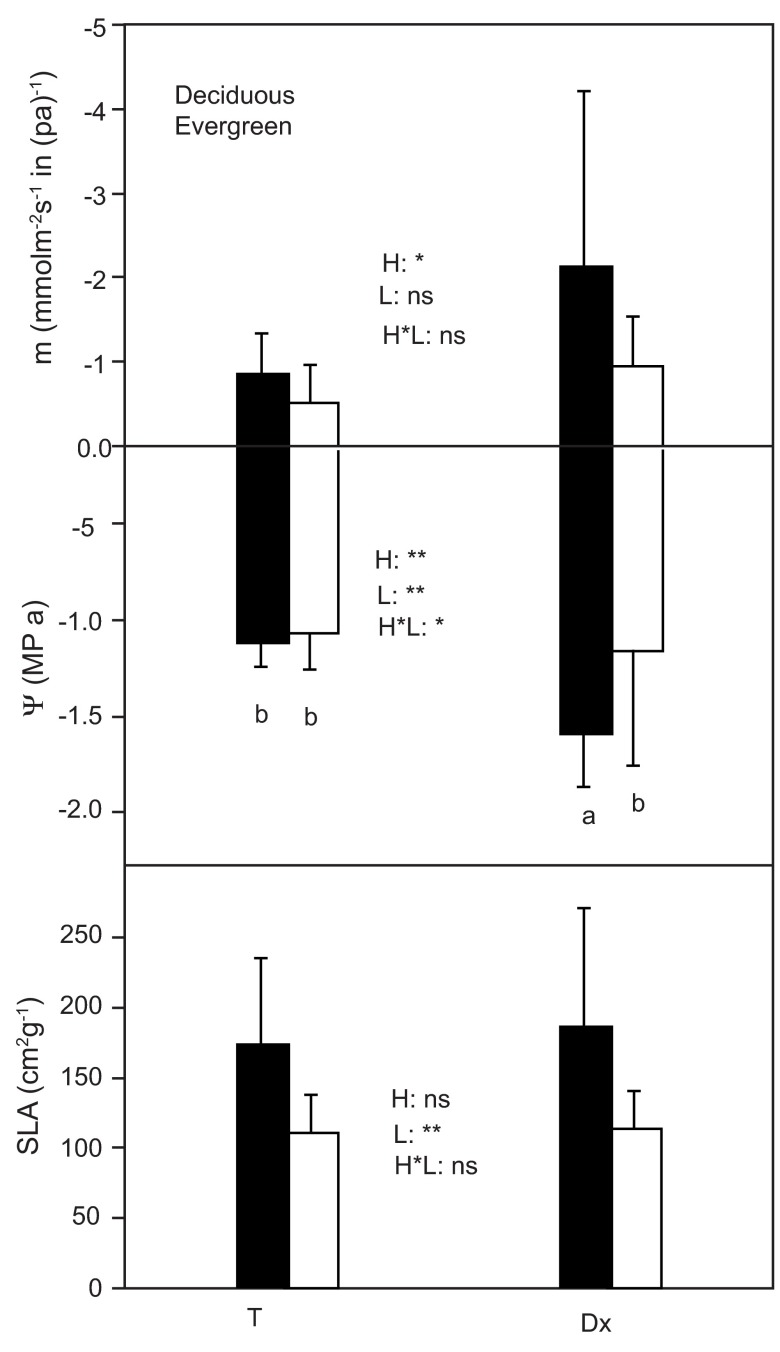
Leaf hydro-physiological and morphological characteristics. Effects of habitat (H), leaf phenology (L), and their interaction (H × L), on stomatal sensitivity (m), leaf water potential (Ψ) and specific leaf area (SLA) (mean ± SD, n = 3–5) of the tree species in two habitats. The ANOVA for Ψ is based on log-transformed data. See Figure 1 for other explanations.

Habitat had a significant effect on stomatal sensitivity (m) (P < 0.05), and the stomatal sensitivity (m) was much higher in Daxiagu than in Tianlongshan ( Figure 3). There was an interactive effect of habitat and leaf phenology on leaf water potential, and the leaf water potential of deciduous tree species in Daxiagu was significantly lower than the leaf water potential in Tianlongshan ( Figure 3). Leaf phenology had a significant effect on the specific leaf area (SLA): the SLA of deciduous species was significantly (P < 0.05) higher than that of evergreen species ( Figure 3).

### Leaf nutrients and their efficiencies for photosynthesis

**Figure 4.**
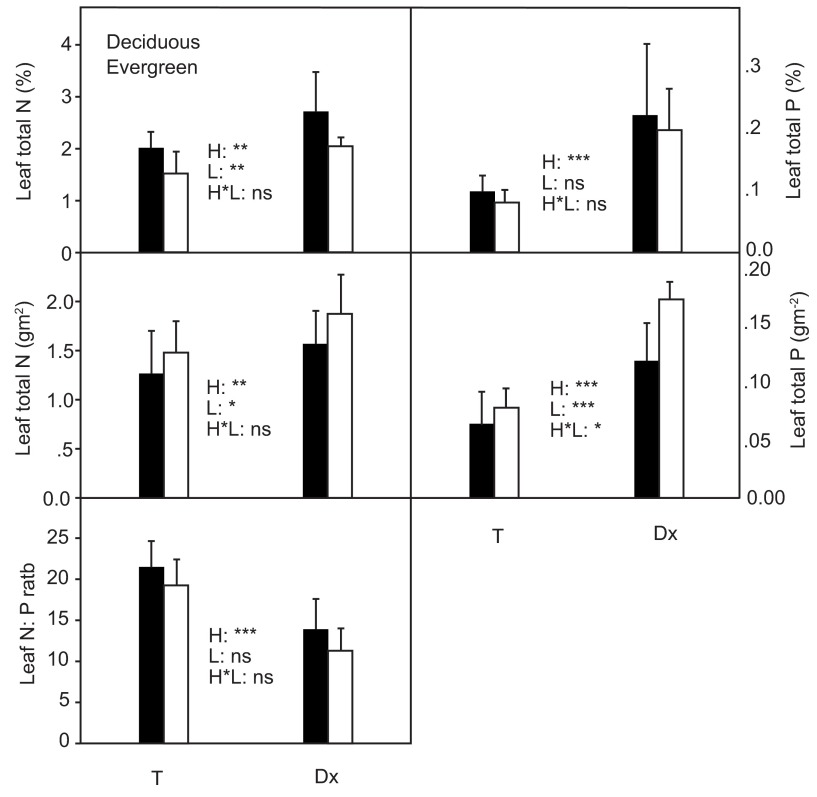
Leaf nutrient status. Effects of habitat (H), leaf phenology (L), and their interaction (H × L), on leaf area based- and mass-based total nitrogen (N) and phosphorus (P) (mean ± SD, n = 3–5) of the tree species in two habitats. See Figure 1 for other explanations.

**Figure 5.**
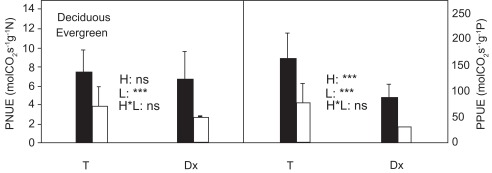
Photosynthetic N and P use efficiency characteristics. Effects of habitat (H), leaf phenology (L), and their interaction (H × L), on photosynthetic N and P use efficiency (PNUE and PPUE, respectively) (mean ± SD, n = 3–5) of the tree species in two habitats. See Figure 1 for other explanations.

Habitat had significant effects on leaf total N content expressed on the basis of either dry mass or leaf area (P < 0.01) ( Figure 4). Leaf total nitrogen content in Daxiagu was much higher than leaf total nitrogen content in Tianlongshan. Leaf phenology also had a significant effect on leaf total nitrogen content expressed on the basis of either dry mass or leaf area (P < 0.01 and P < 0.05, respectively). The mass-based leaf total N content of the deciduous species was much higher than that of the evergreen species. However, when leaf total nitrogen content was expressed on the basis of leaf area, the leaf total N content of the evergreen species was significantly higher than the leaf total N content of the deciduous species. The habitat had a significant effect on leaf total phosphorus content expressed on the basis of either dry mass or leaf area (P < 0.001). The leaf total phosphorus content in Daxiagu was much higher than leaf total phosphorus content in Tianlongshan ( Figure 4). However, when leaf total phosphorus content was expressed on basis of leaf area, the values of the evergreen species were much higher than those of deciduous species, especially in Daxiagu. The habitat also had a significant effect on the ratio of leaf total N to P (N:P) for all measured tree species. The N:P ratio in Tianlongshan was significantly (P < 0.001) higher than the N:P ratio in Daxiagu ( Figure 4). Leaf phenology had a significant (P < 0.001) effect on photosynthetic nitrogen use efficiency (PNUE), and the PNUE of deciduous species was much higher that of evergreen species ( Figure 5). Both habitat and leaf phenology had significant (P < 0.001) effects on photosynthetic phosphorus use efficiency (PPUE). The PPUE of the woody species in Tianlongshan was much higher than that in Daxiagu (P < 0.001), and the PPUE of deciduous species was much higher than that of evergreen species (P < 0.001) ( Figure 5).

Water potential data Leaf water potential of the dominant tree species at Daxiagu and Tianlongshan was measured in the summer season of 2009.

Leaf nutrient and specific leaf area data Mass- and area-based nitrogen and phosphorus content, and specific leaf area of the dominant tree species at Daxiagu and Tianlongshan.

## Discussion

Plants in karst regions are thought to be subject to temporary drought stress in their growth seasons due to the poor water holding capacity of the soil. This is thought to be particularly so in severely degraded karst habitat ^2, 3^. However, the data from this study for chlorophyll fluorescence (e.g. (Φ(Po)), Φ(Eo), and PI(abs)) in the three consecutive growth seasons suggests that drought stress does not occur in all of the investigated tree species during their growth seasons. The chlorophyll fluorescence technique has been used as a powerful tool to assess plant vitality in response to environmental stresses ^10^. So-called "temporary drought stress" may not occur at all. Further, if such temporary stress really does occur, then it may not be severe for most woody plants in the karst areas of western and southwestern Guizhou Province. Further, the indigenous/native trees adapt well to their habitats, including plants in the most severely degraded karst habitat (Daxiagu in this study). This might be due to karst plants’ adaptive structural strategies (e.g. absorbing water from deep soil through roots penetrating into rock crevasses, according to Zhu ^2^), or the plants’ adaptive functions (e.g. stomatal sensitivity to changed environments). It may also be due to the ample precipitation found in the studied areas (annually around 1000 mm, mainly occurring during the growth season). Liu *et al.* ^11^ did find that soil water stress (withholding water) affected photosynthesis and growth, and re-watering could remove or alleviate these effects in potted tree seedlings from almost the same karst area as in our study. However, the water stress treatment (withholding water) period in Liu’s *et al.* experiment was as long as 20 days. According to Zhu *et al.* ^12^, during the growth season, the most common period between two precipitation events in this region is less than 20 days. Further, the available soil water from a heavy rain event could meet tree transpiration needs for 7–14 days ^13^. This implies that in most cases, temporary soil water stress events would not occur during the growth season of this region. The data for leaf δ ^13^C value from Yang *et al.* ^14^ and Fan *et al.* ^15^ also confirms the above conclusions based on chlorophyll fluorescence analyses. They report that the average leaf δ ^13^C values of more than 50 tree species in three karst sites across Guizhou are -27.63% ^14^, and -28.14% ^15^. These values are only higher than the values of the tree species of tropic rain forests in Yunnan Province (e.g. -33.11% reported by Qu *et al.* ^16^), and mostly lower than other areas of China (e.g. -26.24% of temperate forest ^17^ and -27.00% of desert vegetation ^18^). This indicates that the average long-term WUE in tree species in the karst area is low, and that they do not experience severe long-term drought stress.

Nevertheless, we did find a difference in photochemical traits of PSII (i.e. Φ(Po), Φ(Eo), and PI(abs)) between the deciduous and evergreen tree species in the 2009 growth season. This is consistent with the results of midday leaf water potential ( Figure 3), suggesting that the capacity for maintaining leaf hydro-physiological function in evergreen tree species was higher than that in deciduous trees. Fan *et al.* ^15^ also confirmed that evergreen tree species had higher water stress tolerance for maintaining branch hydraulic conductivity than did deciduous trees species. This is based on data for the branch’s hydraulic characteristics for roughly the same tree species in the two habitats. In addition, there was a much lower Ψ50 (xylem tensions at 50% of loss in hydraulic conductivity) in evergreen tree species than in deciduous tree species ^15^.

WUE is an intrinsic trait that indicates plant strategies for environmental adaptation, and is understood in terms of a trade-off between carbon gain and water loss. WUE is also a reliable indicator for determining plant survivorship in arid areas ^19^. To adapt to changed environments, in comparison with plants with low VPD, plants with high leaf-atmosphere VPD will generally increase their WUE ^20, 21^. That there is no significant variation in the effects of habitat and leaf phenology on WUE also partly confirms that plants adapt well to their environments, even to severely degraded karst habitat. Furthermore, our data on photosynthetic gas exchange showed that the photosynthetic rates (Pn) of the tree species in Daxiagu (the severely degraded habitat) are nearly 2–3 times higher than those of the tree species in Tianlongshan (the well developed secondary forest habitat) under ambient conditions. This indicates that to determine the CO _2_ assimilation of plants in these areas, it would be more important to take light and temperature into consideration than it would be to consider precipitation. The Daxiagu habitat has much less vegetation coverage than the Tianlongshan habitat. The tree species in Daxiagu are distributed sparsely and most tree canopies receive almost full sunlight. The tree canopies in Tianlongshan are closed and the light environment is lower for the middle and lower canopy leaves. The temperature in Daxiagu in the growth season is clearly higher than in Tianlongshan (with a difference of about 3–5, and -28.14°C). Many studies confirm that leaves exposed to sunlight have higher Rubisco activity, chlorophyll *a*/ *b*ratio, maximum photosynthetic rate, and light saturation points than do shaded-leaves ^22– 25^. In other words, when precipitation (water supply for soil) is guaranteed, full sunlight and higher temperatures improve the photosynthetic capacity of the plant.

In China, soil phosphorus deficiency occurs more commonly in the southern region than in the northern region. Furthermore, soil total phosphorus density in tropical and subtropical areas is much lower than in other areas ^26^. Soil phosphorus deficiency has a negative effect on plant absorption of nitrogen from the soil; thus phosphorus limitation inhibits plant growth. According to the stoichiometric relationship between N and P, the biomass N:P ratios could be effective indicators of the status of nitrogen and phosphorus in a plant ^27– 29^. The average N:P ratio for terrestrial plant species in their natural field habitats is 12–13 ^30– 32^. Koerselman and Meuleman ^28^ suggest that phosphorus deficiency occurs when the N:P ratio is higher than the critical value of 16. The much higher leaf N:P ratio in Tianlongshan (mean=20.32) as compared to Daxiagu (mean = 12.26) suggests that there was severe phosphorus limitation in Tianlongshan. Because phosphorus deficiency has negative effects on nitrogen absorption ^33, 34^, phosphorus limitation could partly explain why the Pn was lower in Tianlongshan than in Daxiagu, while PPUE was more stimulated in Tianlongshan than in Daxiagu. There is also a marked difference in tree species composition between the two habitats: more than 90% of the tree species measured in Daxiagu are deciduous, while evergreen tree species account for 50% of the measured tree species in Tianlongshan. A great deal of data has shown that the photosynthetic capacity of deciduous species is higher than the photosynthetic capacity of evergreen species ^25^.

The stomatal sensitivity of woody species in open habitats (i.e. Daxiagu) is much higher than in closed habitats (i.e. Tianlongshan), again suggesting that the light, ambient humidity, and temperature conditions are much more important than precipitation in shaping the stomatal response of woody plants to changed leaf-air VPD. The study of the sensitivity of stomata to changed leaf-air VPD conditions in *Ligustrum sinense* also confirmed that the relationship between stomatal conductance and stomatal aperture for high-light leaves was more significant than that of low-light leaves ^35^. Higher stomatal sensitivity will help maintain the physiologically required water status of a plant ^35^. This could explain the difference in the stomatal sensitivity of the woody species between the two habitats. This could also explain why there was no difference in photosynthetic instantaneous WUE between the two habitats, although there was a significant difference in water consumption through transpiration in the woody species.
