# Supplementary material for: Eco-physiological adaptation of dominant tree species at two contrasting karst habitats in southwestern China
Source: F1000Res. 2013 Nov 25;2:122. Originally published 2013 May 9. [Version 2] doi: 10.12688/f1000research.2-122.v2 (PMC3892915; doi:10.12688/f1000research.2-122.v2)
Supplement: Peer review history

# Peer review: Referee response for version 2

**DOI:** 10.5256/f1000research.3069.r2542

**Published:** 2014-07-07

**Authors:** Jessica Gurevitch

**Affiliations:** 1 Department of Ecology and Evolution, Stony Brook University, Stony Brook, NY, USA

This is a very comprehensive ecophysiological study carried out on a wide range of woody species in two contrasting karst sites over the course of three years. The extent and duration of the study is far greater than most comparable field studies, and the paper provides a great deal of valuable information on the physiological responses of woody species in these sites. The differences found between the degraded and intact karst forest must be considered as hypotheses rather than conclusive demonstration of differences between degraded and intact forest, because of the limitations of the experimental design, as discussed in my review of the previous version. Because of the way the study was set up, a more accurate title would be: Ecophysiological responses of dominant tree species in two karst forest sites in southwestern China. I understand that this paper represents an enormous amount of work; ideally, the experimental design could have been thought through with respect to being able to test the factors most of interest before initiating all of that excellent work. In addition, the study design  is not a factorial ANOVA; it is a hierarchical design with a nested structure and thus should have been analyzed as such (thereby changing the F tests and P levels); the reported values for the ANOVA are therefore inaccurate.

I have read this submission. I believe that I have an appropriate level of expertise to confirm that it is of an acceptable scientific standard, however I have significant reservations, as outlined above.

# Response

**Published:** 2014-07-17

**Authors:** Shouren Zhang

**Affiliations:** Chinese Academy of Sciences, China

We sincerely thank Prof. Jessica Gurevitch for her new comments on our revised version. We would like to accept Prof. Jessica Gurevitch’s advice and pay attention on the experimental design in our future work. In response to Prof. Jessica Gurevitch’s comments on statistical issues, we will check our data and calculations, and may update the statistical results later. Regarding the article title, Prof. Jessica Gurevitch suggested to replace eco-physiological adaptation with eco-physiological response. The trees we used for measurements were mature ones which had been naturally grown there more than 20 years old, so the differences in trees’ eco-physiological traits between the two types of karst forests could reflect the variations of the trees’ adaptation to their local habitats. Anyway, we would like to change our mind in this regard if more convincing reasons are kindly provided.

Again, we thank Prof. Jessica Gurevitch for her new comments and suggestions.

# Peer review: Referee response for version 2

**DOI:** 10.5256/f1000research.3069.r2700

**Published:** 2013-12-09

**Authors:** Reto Strasser

**Affiliations:** 1 Professor Emeritus for Bioenergetics, University of Geneva, Geneva, Switzerland

This revised version is in my opinion fully acceptable. The authors have followed the recommendations of the reviewers, and I’ve no additional comments.

I have read this submission. I believe that I have an appropriate level of expertise to confirm that it is of an acceptable scientific standard.

# Peer review: Referee response for version 1

**DOI:** 10.5256/f1000research.1217.r2396

**Published:** 2013-11-13

**Authors:** Reto Strasser

**Affiliations:** 1 Professor Emeritus for Bioenergetics, University of Geneva, Geneva, Switzerland

I read this manuscript several times with great interest. Immediately the reader gets the impression of a carefully conducted and complex investigation. The topic is a case study of ecophysiology as you find it *in vivo*, *in situ* in nature.

The title alone informs the reader that a complex multiparametric ecological experiment is presented.

The areas of this investigation were forests in southwestern China with contrasting habitats, in the Guizhou Province called Tianlongshan and Daxiagu.

Some of the main contrasts are listed and compared below:

- **Altitude above sea level:** 1200m (Tianlongshan) and 900m (Daxiagu)
- **Karst:** Well-developed secondary forest with a humid monsoon climate (Tianlongshan) and severely degraded mixed forest with warm temperatures (Daxiagu)
- **Precipitation:** 1398 mm/year (Tianlongshan) and 1100mm/year (Daxiagu)
- **Rain during growth season:** 60% to 70% (Tianlongshan) and 83% (Daxiagu)
- **Vegetation:** Well developed secondary evergreen and deciduous (Tianlongshan) and secondary deciduous trees and shrubs (Daxiagu)

Branches were cut from both places and used for measurements such as gas exchanges (CO _2_), stomatal conductance, transpiration and water use efficiency (WUE), on new fully expanded leaves.  Many *in vivo in situ* fast fluorescence transients were collected and analyzed according to the equations of the JIP test, which reveals the bioenergetics constellation of the samples.

It is seldom that a research team is able to use so many quite different techniques on the same plant material within a short time span and outside in the forest. All techniques are precisely presented in the manuscript and the execution of the measurements appears to have been done very carefully.

Several years ago I had the opportunity to accompany a research group of the same institute as a guest for another project, to make such measurements in the forest. I became aware of the high professionalism of experimentation which was invested to get the experimental data recorded. E.g. black socks or sock-shaped towels were used to slip over branches on the trees to guarantee dark adaptation in a non-invasive way during the daytime.

In short, I can say that the investigation is technically very complex, however has been executed with a lot of skill and imagination.

I have seen the comments of the previous two reviewers. I fully agree with the scientific statements given by the reviewers who have pointed out the high quality of the data. For some logically understandable reasons one reviewer has given the article a ‘Not approved’ rating. Formally that reviewer is right, that some comparisons bypass strict rigor due to the chosen forest constellation which we cannot change. Most probably there is no better place available for performing these experiments in that area. For ecological investigations we are often forced to select a good site, but even so not all parameters may be optimal. Nevertheless the challenge is to make valid deductions of a non-complete or non-perfect data set. My saying is: A Scientist is allowed to do whatever he likes, as long he is saying what he is doing and how he is doing it. Often even incomplete information provides good hints for a better understanding of the topic. With many repetitions of the measurements and the testing of many samples the authors take care regarding the heterogeneity of the plant material, and minimize the risk of making important errors. My impression is that in the given situation the authors are fully aware about the strong and less strong results or conclusions they are giving in the manuscript.

It needs courage, skill and perseverance to do these experiments and to present the experimental data in the form given in this article. It’s an excellent investigation, written in a well balanced form, based on solid data. All these facts together are arguments to conclude that the manuscript deserves to be approved but the authors should take into account some modifications proposed by the reviewers.

For better understanding I’m proposing some minor changes at the end of the materials and methods section:

- The JIP test parameters used in this study can be summarized as follows:

1. As flux ratios or quantum yields expressed as maximal trapping flux ( **TR _o_**) to the reaction center of PS II, or as maximal electron transport flux ( **ET _o_**) between PS II and PS I, or as maximal heat dissipation flux ( **DI _o_**) of PSII per the light flux absorbed ( **ABS**) by PS II antenna.
2. As partial and cumulated performances (or so called driving forces DF = log of the performances derived in analogy to the redox potentials **.**).

- Please make the frame of Figure 1 taller, so that the labeling of the ordinates on the graphs is better readable and not squashed.
- The partial yields or probabilities or performances are based on the functional flux-ratios and can be correlated to the following experimental signals:                                       

**F** **_P_** **_o_** **= **TR _o_ /ABS = 1-Fo/Fm = Fv/Fm

**y** **_E_** **_o_** =  ET _0_ /TR _o_  = (1-V _J_ ) where V _J_ = (F _J_ – Fo)/(Fm – Fo)  is the relative variable fluorescence at the J-step, and the structural ratio:

**g** **_RC_**  =   Chl _RC_ / Chl _total_ = Chl _RC_ / Chl _(RC+Antenna)   _or     

g _RC _/(1-g _RC _) = Chl _RC_ / Chl _Antenna _ = RC/ABS = 1/ Antenna size of PS II

Quantum yield (this means: per absorption ABS) for primary photochemistry **:** 

**F** **_P_** **_o_** **= **TR _o_ /ABS

Quantum yield for electron transport **:**

**F** **_E_** **_o_** **=** F _P__o __* _y _E__o_ **= **ET **_o_** **/ **ABS as the probability that a photon absorbed by PS II will move an electron into the electron transport chain further than Qa ^-^.

Quantum yield for heat dissipation by PS II: 

**F** **_D_** **_o_** = 1 - F _P__o _ = DI _o_ /ABS   (please use y _E__o_   to distinguish it from y, which you are using for the midday leaf water potential.)

The performance index **PI _ABS_**, which is responsible for the performance of the electron transport from water to plastoquinone is written as:

**PI _ABS_** = ((g _RC _/(1-g _RC_)) x ((F _P__o _/ (1-F _P__o_)) x ((y _E__o_ /(1- y _E__o_))

The multi parametric approach to this realistic field study is appreciated and proves that the authors have a good ecological feeling and understanding.

I have read this submission. I believe that I have an appropriate level of expertise to confirm that it is of an acceptable scientific standard.

# Peer review: Referee response for version 1

**DOI:** 10.5256/f1000research.1217.r1026

**Published:** 2013-06-26

**Authors:** Jessica Gurevitch

**Affiliations:** 1 Department of Ecology and Evolution, Stony Brook University, Stony Brook, NY, USA

Scientific study of the ecologically interesting karst forests of southwestern China has been limited, particularly in the English-language literature, making this study potentially valuable. The comparison of *in situ* with common environment performance was excellent. However, major design flaws make it impossible to reach scientifically valid conclusions from this study. Most importantly, the study compares one degraded forest with one more-intact forest in order to reach conclusions about the ecophysiology of trees in degraded karst forests in comparison to intact forests. This is not possible with one forest of each type; the fallacy in this kind of comparison has been highlighted in the ecological literature for decades (sometimes called “pseudoreplication”). One cannot reach any valid conclusions from comparing trees in a single forest of one type with those of a single forest of a different type. The anova also fails to account for the nested design structure, a very common problem in the ecological literature.

I have read this submission. I believe that I have an appropriate level of expertise to state that I do not consider it to be of an acceptable scientific standard, for reasons outlined above.

# Response

**Published:** 2013-07-09

**Authors:** Shouren Zhang

**Affiliations:** Chinese Academy of Sciences, China

We appreciate the invaluable comments from Prof. Jessica Gurevitch. We agree with Prof. Gurevitch’s comments on the experimental design in our study. However, at least two factors prevented us from selecting more sites for each forest type. Firstly, field acquisition of physiological parameters, such as the photosynthetic rate and maximum photochemical efficiency of PSII, is very time-consuming. Our ability to make the simultaneous measurements needed to compare different sites is therefore limited by resource constraints. Secondly, since these physiological parameters vary significantly throughout the day, they need to be measured at similar times of day to make sense of the comparison. For example, in this study we measured photosynthetic gas exchange and chlorophyll fluorescence from late-morning to noon, which is thought to be the daily based maximal photosynthetic functional period for the sampled trees. These factors make physiological investigations under controlled conditions on multiple sites unpractical. We note, also, that although we compared only two sites, the trees sampled within one site in our study were distributed over a large area (roughly 1 km ^2^), and therefore could be regarded as growing on different sub-sites with quite different local environmental situations. Most importantly, however, the physiological data in our study were collected over three consecutive years in field. Such long-term investigations of physiological processes in field are very scarce in eco-physiological literature.

# Peer review: Referee response for version 1

**DOI:** 10.5256/f1000research.1217.r968

**Published:** 2013-05-29

**Authors:** Jian Rang Wang

**Affiliations:** 1 Faculty of Natural Resources Management, Lakehead University, Thunder Bay, Canada

The title is appropriate for the content of the article. The authors used two contrasting habitats (degraded versus well developed) and leaf phenology (deciduous versus evergreen) across many major tree species to address the ecophysiological adaptation of different tree species to habitat change. The research was well designed and relatively long-term (three year measurements) for ecophysiological study. The information presented in the study will provide significant insight to our understanding of tree species adaptation to climate change and habitat degradation. The conclusions from the study will provide useful guidance for biodiversity management and restoration of degraded sites.

I have read this submission. I believe that I have an appropriate level of expertise to confirm that it is of an acceptable scientific standard.
